# Optically sizing single atmospheric particulates with a 10-nm resolution using a strong evanescent field

**DOI:** 10.1038/lsa.2018.3

**Published:** 2018-04-20

**Authors:** Xiao-Chong Yu, Yanyan Zhi, Shui-Jing Tang, Bei-Bei Li, Qihuang Gong, Cheng-Wei Qiu, Yun-Feng Xiao

**Affiliations:** 1 State Key Laboratory for Mesoscopic Physics and School of Physics, Peking University, Beijing 100871, China; 2 Collaborative Innovation Center of Quantum Matter, Beijing 100871, China; 3 Collaborative Innovation Center of Extreme Optics, Shanxi University, Taiyuan 030006, China; 4 Department of Electrical and Computer Engineering, National University of Singapore, Singapore 117583, Singapore; 5 NUS Suzhou Research Institute (NUSRI), Suzhou 215123, China; 6 SZU-NUS Collaborative Innovation Center for Optoelectronic Science & Technology, Shenzhen University, Shenzhen 518060, China

**Keywords:** air pollution, optical nanofiber, single particulates, size spectrometer

## Abstract

Although an accurate evaluation of the distribution of ultrafine particulate matter in air is of utmost significance to public health, the usually used PM_2.5_ index fails to provide size distribution information. Here we demonstrate a low-profile and cavity-free size spectrometer for probing fine and ultrafine particulate matter by using the enhanced particle-perturbed scattering in strong optical evanescent fields of a nanofiber array. The unprecedented size resolution reaches 10 nm for detecting single 100-nm-diameter nanoparticles by employing uniform nanofibers and controlling the polarizations of the probe light. This size spectrometry was tested and used to retrieve the size distribution of particulate matter in the air of Beijing, yielding mass concentrations of nanoparticles, as a secondary exercise, consistent with the officially released data. This nanofiber-array probe shows potential for the full monitoring of air pollution and for studying early-stage haze evolution and can be further extended to explore nanoparticle interactions.

## Introduction

The global environment is suffering from air pollution due to excess particulate matter, resulting in huge societal and economic costs^1, 2, 3, 4, 5^. Air quality is usually characterized by the mass concentration of fine particulate matter with aerodynamic diameters <2.5 μm (PM_2.5_), which is mainly contributed by micron-sized particles, whereas the hazard induced by ultrafine particulates (with diameters smaller than hundreds of nanometers) remains seriously underestimated. For instance, ultrafine particles are believed to have even more aggressive health implications than larger particulates because they can penetrate the lungs, causing lung cancer, and can also penetrate the air–blood barrier^6^, entering the circulation system and resulting in respiratory illness and even organ dysfunction^7, 8, 9, 10, 11, 12, 13^. Therefore, more attention should be paid to the ultrafine particles, and their size distribution, in addition to their mass distribution, is becoming increasingly important for the evaluation of air hazards.

**Figure 1:**
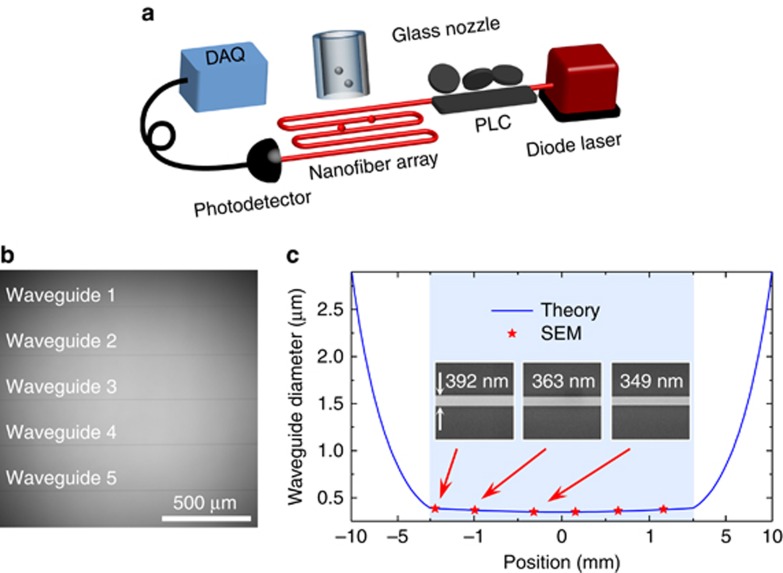
Size probing system. (**a**) Schematic set-up of nanowaveguide-based size spectrometry. The analyte nanoparticles are blown onto the nanowaveguides via a glass nozzle. DAQ, data acquisition system; PLC, polarization controller. (**b**) Optical image of the nanowaveguides, consisting of five in-serial identical nanofibers with a distance of 250 μm between each adjacent two. (**c**) Comparison of the diameter distribution of a nanowaveguide from the theoretical prediction (blue curve) and scanning electron microscopic (SEM) measurements (red stars). Inset shows SEM images of three segments, with diameters of 392, 363 and 349 nm from left to right. The shadow marks the waveguide range with the length of 3 mm, for which the diameter variation is approximately 10%. Note that the scale of the horizontal axis in the range of −1.5 to 1.5 mm is different from the other range.

Various techniques have been developed for measuring the size distribution of particulate matter. Compared with the conventional aerosol techniques such as the use of a scanning mobility particle sizer, an electrical low pressure impactor and a tapered element oscillation microbalance^14, 15^, optical methods show great potential due to their non-destructive nature, electromagnetic noise immunity and real-time *in situ* detection capability^16, 17, 18, 19^. The typical optical methods mainly utilize absorption or scattering methods. However, the absorption methods are only applicable for lossy targets^20, 21, 22^, while the scattering methods, including dynamic light scattering and static light scattering^23, 24^, either require ensemble analytes with high enough concentrations^25, 26, 27, 28^ or suffer from low accuracy in particle size estimation^29, 30, 31^. In addition, the conventional scattering methods using free space laser light must be operated in a closed cavity to avoid disturbance from environmental light, thus making the system rather complicated. Interferometric scattering microscopy and photothermal microscopy have both achieved single nanoparticle detection^32, 33, 34^ but require a highly stable light source and extra imaging systems, thus also making the detection system quite complicated. The recently developed optical microcavity sensing systems^19, 35, 36, 37, 38, 39, 40, 41, 42, 43, 44^ using scattering methods have removed the requirement for a closed cavity and achieved an unprecedented low detection limit. However, microcavity-based sizing typically requires a tunable laser source and the strict control of near-field coupling. In this work, we propose and demonstrate a low-profile, high-accuracy, cavity-free and real-time size probing system working in an open environment using a nanowaveguide array structure with a strong evanescent field (Figure 1a and 1b). The spectrometry capability of the new system is first characterized by measuring the size distribution of single standard polystyrene (PS) nanoparticles, and a sizing resolution of 10 nm is achieved for 100-nm-diameter nanoparticles by controlling the polarizations of the probe light. Then the size spectrometer is applied to monitor the fine and ultrafine particulate matter in Beijing from 11 December 2015 to 12 January 2016 and from 25–26 December 2016 when the first red alert on air pollution was issued in Beijing. In addition, the mass concentrations are obtained from the size information on the size distributions, for which the evolution trend agrees with the official data presented by the Beijing Municipal Environmental Monitoring Center (see Supplementary Information).

## Materials and methods

### Fabrication of the nanowaveguide structure and the nozzle

To increase the sensing area, we fabricated a nanowaveguide structure consisting of five nanofibers instead of one (see Figure 1a) with a long joint fiber loop to minimize the polarization change. A single fiber arranged into a serpentine pattern with five turns is pulled at the same time using a home-built heat-and-pull system containing a ceramic microheater (NTT-AT, CMH-7022, Kawasaki, Japan) and a step motor (Shanghai-Lianyi, Model XXM80H-50, Shanghai, China). The temperature of the heater is set to be >1200 °C over the length of 9 mm to obtain a nanofiber of uniform diameter along the length of several millimeters. By controlling the fabrication parameters, such as the heating temperature, the pulling rate and the pulling time period, the nanofibers can be repeatedly fabricated with reproducible diameters. A glass nozzle is fabricated by heating and pulling a glass capillary to a final inner diameter of ∼50 μm using the same microheater and the step motor.

### Particle preparation

The standard PS nanoparticles (Thermo Fisher Scientific) used as the benchmark analytes are first diluted to tens of picomoles and then atomized using an ultrasonic atomizer to prevent particle aggregations. As the ultrafine particulate matter in the air samples is examined, a filter membrane is used in front of the syringe to eliminate the influence of the particles with diameters >1.0 μm. The Rayleigh–Gans theory can thus be applied to calculate the particle size (see Section I of Supplementary Information).

### Measurement system

A syringe pump (Harvard, Model PHD22/2000) is used to inject the nanoparticles into a gas pipe and then to the nanowaveguides via the glass nozzle. The probe light source is a 680-nm diode laser (New Focus, Model TLB6309), and a photodetector (Newport, Model 918D) is used to monitor the transmitted power in real time. A polarization controlling system consisting of a quarter wave plate, a polarizer and a polarization controller was built to generate a circular polarized probe light.

### Data acquisition and analysis

The transmission power collected by the photodetector is analyzed by a data acquisition system (National Instruments, Model USB-6251 BNC) with an acquisition rate of 100 kS s^−1^. A 2000-point average is first applied to suppress the relative noise level (i.e., the ratio of the noise of the power to the absolute power) from 10^−3^ to ∼10^−4^. A step-finding algorithm is applied to determine the step drops in the transmission power following Yu *et al*^45^, corresponding to the single nanoparticle-binding events on the nanowaveguide.

## Results and discussion

The nanowaveguide structure consists of serpentine patterned nanofibers (with a 250-μm separation between each two adjacent nanofibers), fabricated using a home-built heat-and-pull system including a ceramic heater and a step motor (see Supplementary Information and Refs. 46, 47). The optical image is shown in Figure 1b. As the heating temperature is highly uniform in a large area, the five nanowaveguides are almost identical and the diameter of each one is uniform within the range of a few millimeters, thus significantly decreasing the uncertainty of the nanoparticle sizing measurements. Experimentally, the diameter of the waveguides is determined by controlling the elongation length and the heating temperature. The profile of the resulting nanowaveguides can be predicted using the thin filament equation (the blue solid curve in Figure 1c), which is confirmed by the results from the scanning electron microscopic measurements (the red stars in Figure 1c). These result shows that the size variation of a nanowaveguide (with a waist diameter of ∼350 nm) is ∼10% within the 3-mm length, confirming the reasonable uniformity of the sensing area.

The analyte nanoparticles are blown onto the nanowaveguide structure using a glass nozzle (shown in Figure 1a) via a syringe pump (see Supplementary Information). The flow rate is set at 20 mL min^−1^, so that individual nanoparticles bind on the waveguide separately, and the multi-particle-binding event probability is <2% (see Supplementary Information). A 680-nm wavelength diode laser is used as the probe light source, and the transmitted power is monitored by a photodetector in real time. The transmission change transduces the particle-induced scattering, providing the information on the nanoparticle size.

**Figure 2:**
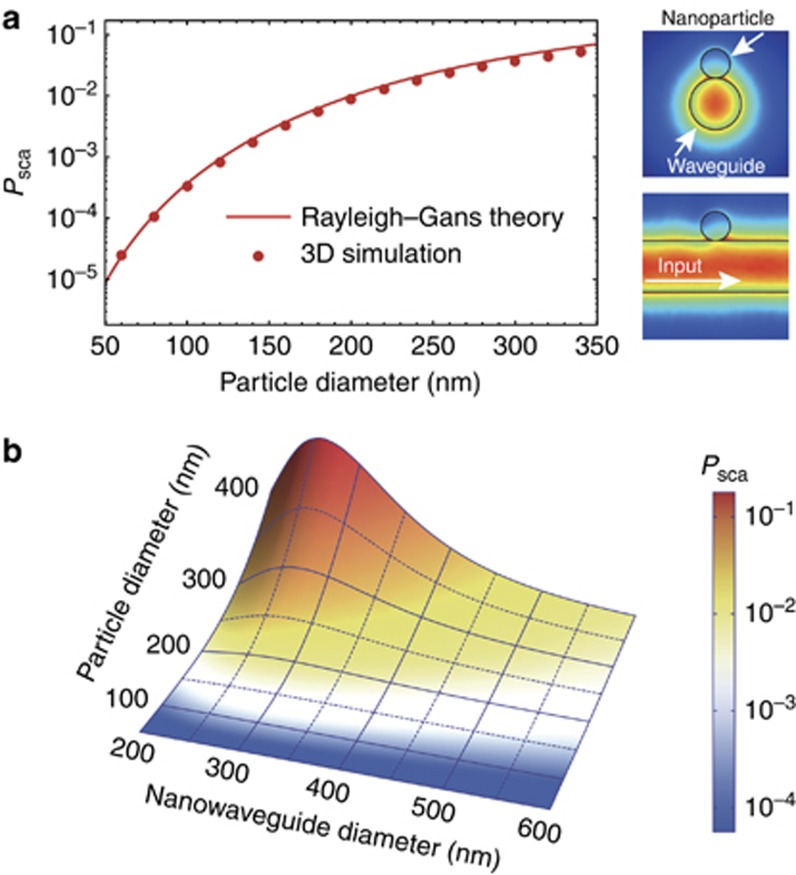
Simulation results based on Rayleigh–Gans theory. (**a**) Scattering efficiency as a function of the particle diameter calculated by Rayleigh–Gans theory (solid curves) and three-dimensional finite-element-method (FEM) simulation (symbols). The diameter of the nanowaveguide is 350 nm. The operation wavelength is 680 nm. The refractive indices of the nanowaveguide and the nanoparticle are 1.46 and 1.5, respectively. The inset shows the FEM simulated results for the electric field distribution around (up right) and along (bottom right) the waveguide for circularly polarized light when a 200-nm-diameter PS nanosphere binds to a 350-nm-diameter nanowaveguide. (**b**) Scattering efficiency of a spherical nanoparticle as a function of the waveguide diameter and particle size.

Considering the inhomogeneous evanescent field across the nanoparticle, the Rayleigh–Gans scattering theory (see Supplementary Information) is employed to determine the scattering efficiency *P*_sca_, defined as the scattering power divided by the pump power^45, 48, 49^. The interaction between the nanoparticle and the guided light is in the perturbation regime, and strong coupling is absent^50^. In addition to the transverse fields, the longitudinal field is also considered in the calculation^51^. The scattering efficiency depends on the properties of the nanowaveguide (evanescent field distribution), the analyte nanoparticle (size and refractive index) and the surrounding medium (refractive index). Note that the fundamental HE_11_ modes of a cylindrical dielectric waveguide are degenerate with different polarizations^52^. The field distribution becomes homogeneous in the angular direction only when the probe light is circularly polarized, and the scattering efficiency induced by a nanoparticle in the vicinity of the nanowaveguide does not depend on the binding position, as verified by the finite-element-method simulation results (see the inset of Figure 2a). Using circularly polarized probe light, the solid curve of Figure 2a presents the analytical result for the scattering efficiency *P*_sca_ induced by a single nanoparticle on a 350-nm-diameter waveguide as a function of the nanoparticle diameter. The analytical calculations of scattering efficiencies obtained using the Rayleigh–Gans theory are confirmed by the three-dimensional finite-element-method simulation results (symbols, Figure 2a)^52^.

Using the Rayleigh–Gans scattering method, we further study the scattering efficiency *P*_sca_ as a function of both the nanosphere and nanowaveguide diameters (shown in Figure 2b). As expected, the scattering efficiency *P*_sca_ shows a monotonic dependence on the nanoparticle size for a given nanowaveguide diameter and reaches a maximum at the diameter of ∼250 nm due to the trade-off between the intensity and confinement of the optical evanescent field^53^. In the experiment, we use the waveguide with a diameter slightly >250 nm, such as 350 nm in our case, for the following reasons. First, the waveguide with a larger diameter is more robust and shows higher transmission^54, 55^. Second, a slightly lower scattering efficiency, in fact, allows us to observe more particle-binding events before the waveguide transmission drops to the noise level. In addition, only the fundamental mode is supported in the 350-nm-diameter waveguide, and multiple guiding modes will invalidate the spectrometry performance due to the mode interference. Therefore, we conduct the sensing experiment using nanowaveguides with diameters of ∼350 nm. The scattering efficiency is obtained from the step change in the transmission, and then the size of a single nanoparticle is directly derived from the scattering efficiency using the provided information regarding the nanowaveguide size and index of the nanoparticle.

**Figure 3:**
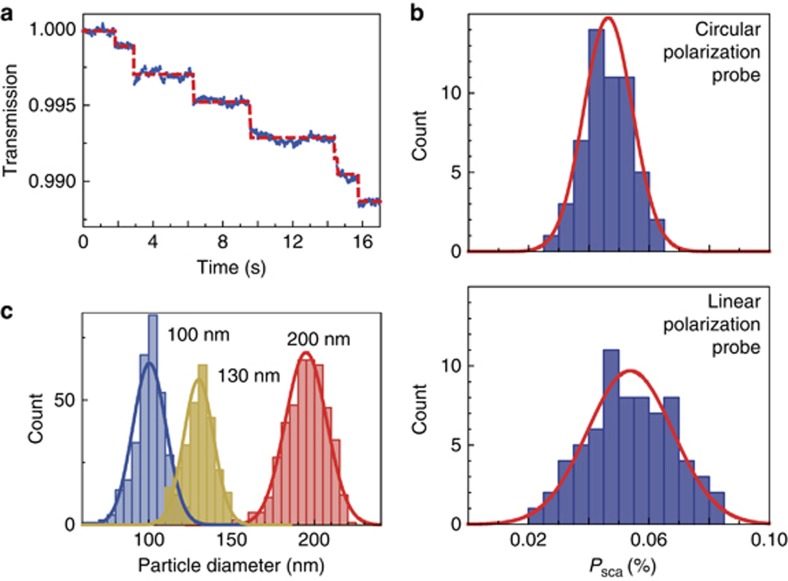
Detection and sizing of standard PS nanoparticles. (**a**) Normalized transmission power when single nanoparticles with a diameter of 130 nm attached to the waveguide (*d*~350 nm). The red dashed curve is plotted to guide the eye. (**b**) Statistical analysis for scattering efficiencies induced by PS nanoparticles with a single diameter of 90 nm using the same nanowaveguide when the probe light is circularly (top panel) and linearly (bottom panel) polarized. Red curves show the Gaussian fitting. (**c**) Statistical analysis for nanoparticle sizes with diameters of 100, 130 and 200 nm using circularly polarized probe light.

A typical transmitted power of the nanowaveguide in real time is shown in Figure 3a for the single PS nanospheres generated using an ultrasonic atomizer binding onto the nanowaveguides (see Supplementary Information). The particle-binding events are clearly recognized by the stepwise drop in the transmitted power. The statistical distribution of the scattering efficiencies for single nanoparticles with a diameter of 89.4±5.3 nm is shown in Figure 3b. It can be seen that the full width at half maximum of the distributions is 0.019% for the circularly polarized light, whereas the full width at half maximum is ∼0.033% for the linearly polarized probe light (see Supplementary Information). The reduced full width at half maximum ensures a lower uncertainty for the size measurement of single nanoparticles. In the following sizing experiments, the circularly polarized light is thus used as a probe. The nanoparticle size distribution is derived from the scattering efficiency distribution, given that the refractive index of the PS nanoparticle is 1.59.

To further evaluate the sizing capability of the nanowaveguide probe, standard PS nanospheres with diameters of 100, 130, and 200 nm are tested. The histograms of the particle diameters obtained using Gaussian fitting show that the measured nanoparticle sizes fall into the ranges of 100±10 nm, 130±12 nm and 200±15 nm for the diameters, indicating the high resolution of the nanowaveguide-based size spectrometry (see Figure 3c). The experimental resolution is limited by the particle size distribution and the particle–particle interaction mediated by the guided mode (see Supplementary Information).

**Figure 4:**
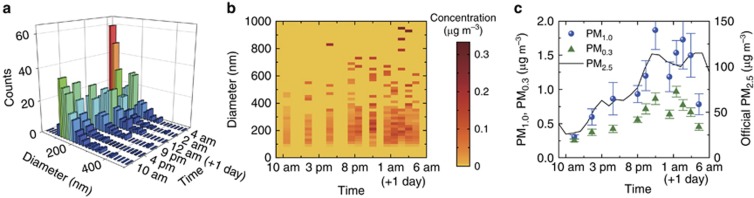
Urban air monitoring from 11 to 12 December 2015. (**a**) Size histogram of nanoparticles in six air samples collected from different moments. (**b**) Evolution of the mass concentration of the nanoparticles of different diameters with a 20-nm step. (**c**) Evolution of the measured PM_1.0_ and PM_0.3_ indices (blue spheres and green triangles, respectively, left axis) compared with the official PM_2.5_ index from Beijing Municipal Environmental Monitoring Center (solid curve, right axis). Error bars indicate the standard deviation.

We then tested our system using local air samples collected in real time from the urban atmosphere in the winter, when the haze problem is most severe. The effective sizes of particulate matter are derived from the scattering efficiency using the Rayleigh–Gans theory (see Supplementary Information), assuming that the refractive index is 1.5 (the particulates in air are mainly nitrates and sulfides, with refractive indices ranging from 1.45 to 1.55). The change in the refractive index will induce a size uncertainty of <10% (see Supplementary Information). For instance, the nanoparticle size is derived to be 235.7±14.5 nm for a measured scattering efficiency of 1%. The derived size distribution of the nanoparticles is plotted in Figure 4a for six typical samples collected from 10 am on 11 December 2015 to 5 am the next day.

Using the size distributions, we also obtain the mass concentration distributions of the nanoparticles with a diameter step of 20 nm, as plotted in Figure 4b. The PM_1.0_ (PM_0.3_) index, defined as the mass concentration of particulate matter with effective diameters <1.0 μm (0.3 μm), is further obtained and reported in Figure 4c. The trend in evolution of the experimental PM_1.0_ agrees with the official PM_2.5_ data from Beijing Municipal Environmental Monitoring Center. The ultrafine particulate matter <300 nm only contributes approximately half of the PM_1.0_ index most of the time, but its counts are dominant, as observed from the size distribution presented in Figure 4a. The size distribution provides information that cannot be obtained from the typical PM_2.5_ (PM_1.0_) index. We note that the measured PM_1.0_ has a different amplitude from the official PM_2.5_ data because the experimental PM_1.0_ index is obtained by analyzing the particle-binding events onto the nanowaveguides, and not all particulate matter in the air samples is detected. Future experiments could estimate the probability of the nanoparticles being captured by nanowaveguides, and the absolute value of the PM_*x*_ index, with *x*=1.0, 0.3 and 0.1, for example, can be provided definitely.

**Figure 5:**
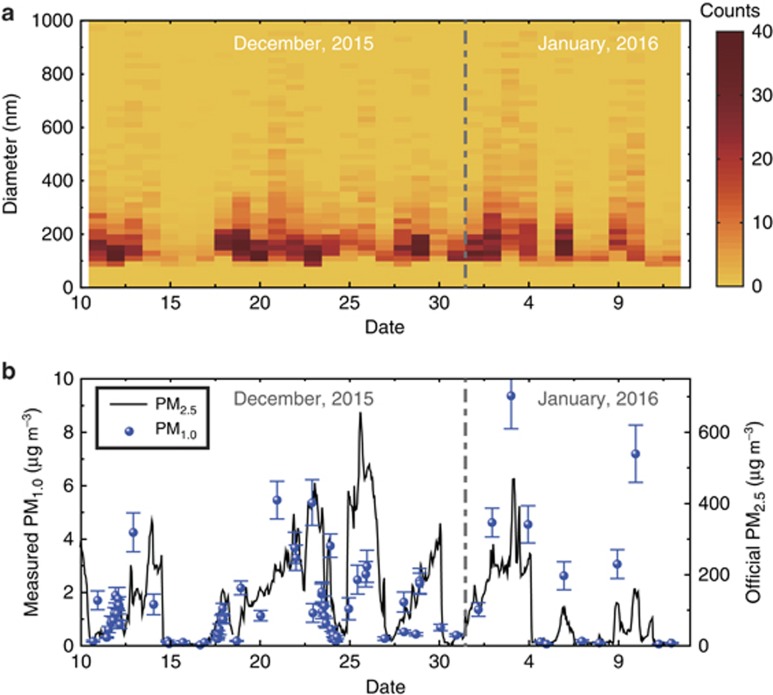
One-month data for PM_1.0_ measured by the size spectrometer. The size distribution (**a**) and the mass concentration (**b**) of the particulate matter from 11 December 11 2015 to 12 January 2016. The symbols and the solid curve indicate the experimental PM_1.0_ data and the official PM_2.5_ data reported by Beijing Municipal Environmental Monitoring Center. The error bars indicate the standard deviation.

The particulate matter in the Beijing atmosphere from 11 December 2015 to 12 January 2016 is monitored, with the size distribution and the mass concentration plotted in Figure 5a and 5b, respectively. On clear days, for example, from 15 to 17 December 2015, the counts of the nanoparticles are quite low (Figure 5a), and the mass concentration remains <50 μg m^−3^ (Figure 5b). On the days with severe haze, such as 3 and 4 January 2016, the counts of ultrafine particles are much higher. The trend in the evolution of the experimental PM_1.0_ results is consistent with that of the official PM_2.5_ data shown in Figure 5b. For instance, there are five peaks in the PM_2.5_ data that are also observed in the experimental PM_1.0_ data. We note that large deviations appear on the haze days because larger particulates contribute to the mass concentration of the PM_2.5_ index, for example, on 26 December. The PM index shows better agreement on clear days, for example, from 15 to 20 December, because smaller particulates are the main contributors to both the PM_1.0_ and PM_2.5_ indices. The particulate matter was also monitored from 25 to 26 December 2016, when the Beijing municipal government issued the first red alert for air pollution in 2016, and the experimental results are also confirmed by the official data (see Supplementary Information).

## Conclusion

To summarize, we have developed a portable, real-time and ultrasensitive nanoparticle size spectrometer with a size uncertainty as low as 10 nm. This cost-effective and reliable probe system was adopted to monitor the ultrafine particulate matter in urban atmospheres, and both the size and mass concentration distributions were obtained, providing important information for environmental monitoring and pollution control. In future experiments, several improvements could be developed. First, a well-designed nozzle will increase the capture rate and decrease the detection time. Second, the sensitivity of a nanowaveguide sensor can be significantly improved by building a ring-structure microcavity, by utilizing plasmonic field localization to enhance the light–matter interaction^19^ or by applying heterodyne interferometry to achieve a quantum noise level^56^. Finally, several light sources can be simultaneously coupled into the nanowaveguide, enabling specific detection of nanoparticles. The nanofiber-array-based probe can further be extended to study nanoparticle interactions and quantum electromagnetic dynamics^38, 57, 58, 59, 60^, and the scattering effects can also be used in microscopic techniques^61, 62, 63, 64, 65^.

## Author contributions

XC Yu and SJ Tang conducted the experiment. XC Yu performed the simulation. XC Yu, YF Xiao and CW Qiu analyzed the experimental data. YF Xiao conceived the idea and supervised the project. All authors contributed to and wrote the paper.

## References

[bib1] Brook RD, Rajagopalan S, Pope CA III, Brook JR, Bhatnagar A et al. Particulate matter air pollution and cardiovascular disease: an update to the scientific statement from the American Heart Association. Circulation 2010; 121: 2331–2378. 20458016 10.1161/CIR.0b013e3181dbece1

[bib2] Burnett RT, Pope CA III, Ezzati M, Olives C, Lim SS et al. An integrated risk function for estimating the global burden of disease attributable to ambient fine particulate matter exposure. Environ Health Perspect 2014; 122: 397–403. 24518036 10.1289/ehp.1307049 PMC3984213

[bib3] Kelly FJ, Fussell JC. Size, source and chemical composition as determinants of toxicity attributable to ambient particulate matter. Atmos Environ 2012; 60: 504–526.

[bib4] Schmale J, Shindell D, von Schneidemesser E, Chabay I, Lawrence M. Air pollution: clean up our skies. Nature 2014; 515: 335–337. 25409811 10.1038/515335a

[bib5] Lelieveld J, Evans JS, Fnais M, Giannadaki D, Pozzer A. The contribution of outdoor air pollution sources to premature mortality on a global scale. Nature 2015; 525: 367–371. 26381985 10.1038/nature15371

[bib6] Oberdörster G, Sharp Z, Atudorei V, Elder A, Gelein R et al. Extrapulmonary translocation of ultrafine carbon particles following whole-body inhalation exposure of rats. J Toxicol Environ Health A 2002; 65: 1531–1543. 12396867 10.1080/00984100290071658

[bib7] Elsaesser A, Howard CV. Toxicology of nanoparticles. Adv Drug Deliver Rev 2012; 64: 129–137. 10.1016/j.addr.2011.09.001 21925220

[bib8] Davidson CI, Phalen RF, Solomon PA. Airborne particulate matter and human health: a review. Aerosol Sci Technol 2005; 39: 737–749.

[bib9] Nel AE, Mädler L, Velegol D, Xia T, Hoek EMV et al. Understanding biophysicochemical interactions at the nano-bio interface. Nat Mater 2009; 8: 543–557. 19525947 10.1038/nmat2442

[bib10] Oberdörster G, Oberdörster E, Oberdörster J. Nanotoxicology: an emerging discipline evolving from studies of ultrafine particles. Environ Health Perspect 2005; 113: 823–839. 16002369 10.1289/ehp.7339 PMC1257642

[bib11] Choi HS, Ashitate Y, Lee JH, Kim SH, Matsui A et al. Rapid translocation of nanoparticles from the lung airspaces to the body. Nat Biotechnol 2010; 28: 1300–1303. 21057497 10.1038/nbt.1696 PMC3058321

[bib12] Arora S, Rajwade JM, Paknikar KM. Nanotoxicology and *in vitro* studies: the need of the hour. Toxicol Appl Pharmacol 2012; 258: 151–165. 22178382 10.1016/j.taap.2011.11.010

[bib13] Underwood E. The polluted brain. Science 2017; 355: 342–345. 28126768 10.1126/science.355.6323.342

[bib14] Ruzer LS, Harley NH. Aerosols Handbook: Measurement, Dosimetry, and Health Effects2nd edn.Boca Raton: CRC Press; 2013.

[bib15] Giechaskiel B, Maricq M, Ntziachristos L, Dardiotis C, Wang XL et al. Review of motor vehicle particulate emissions sampling and measurement: from smoke and filter mass to particle number. J Aerosol Sci 2014; 67: 48–86.

[bib16] Anker JN, Hall WP, Lyandres O, Shah NC, Zhao J et al. Biosensing with plasmonic nanosensors. Nat Mater 2008; 7: 442–453. 18497851 10.1038/nmat2162

[bib17] Fan XD, White IM, Shopova SI, Zhu HY, Suter JD et al. Sensitive optical biosensors for unlabeled targets: a review. Anal Chim Acta 2008; 620: 8–26. 18558119 10.1016/j.aca.2008.05.022 PMC10069299

[bib18] Lou JY, Wang YP, Tong LM. Microfiber optical sensors: a review. Sensors 2014; 14: 5823–5844. 24670720 10.3390/s140405823 PMC4029688

[bib19] Foreman MR, Swaim JD, Vollmer F. Whispering gallery mode sensors. Adv Opt Photonics 2015; 7: 168–240. 26973759 10.1364/AOP.7.000168 PMC4786191

[bib20] Arnott WP, Hamasha K, Moosmüller H, Sheridan PJ, Ogren JA. Towards aerosol light-absorption measurements with a 7-wavelength Aethalometer: evaluation with a photoacoustic instrument and 3-wavelength nephelometer. Aerosol Sci Technol 2005; 39: 17–29.

[bib21] Knittel J, Chow JH, Gray MB, Taylor MA, Bowen WP. Ultrasensitive real-time measurement of dissipation and dispersion in a whispering-gallery mode microresonator. Opt Lett 2013; 38: 1915–1917. 23722788 10.1364/OL.38.001915

[bib22] Shen BQ, Yu XC, Zhi YY, Wang L, Kim DH et al. Detection of single nanoparticles using the dissipative interaction in a high-*Q* microcavity. Phys Rev Appl 2016; 5: 024011.

[bib23] Berne BJ, Pecora R. Dynamic Light Scattering: with Applications to Chemistry, Biology, and Physics. New York: John Wiley & Sons; 1976.

[bib24] Xu RL. Particle Characterization: Light Scattering Methods. Dordrecht: Springer; 2002.

[bib25] Xu RL. Light scattering: a review of particle characterization applications. Particuology 2015; 18: 11–21.

[bib26] Dieckmann Y, Cölfen H, Hofmann H, Petri-Fink A. Particle size distribution measurements of manganese-doped ZnS nanoparticles. Anal Chem 2009; 81: 3889–3895. 19374425 10.1021/ac900043y

[bib27] Fissan H, Ristig S, Kaminski H, Asbach C, Epple M. Comparison of different characterization methods for nanoparticle dispersions before and after aerosolization. Anal Methods 2014; 6: 7324–7334. 10.1039/c4ay01203h 40883942

[bib28] Nobbmann U, Connah M, Fish B, Varley P, Gee C et al. Dynamic light scattering as a relative tool for assessing the molecular integrity and stability of monoclonal antibodies. Biotechnol Genet Eng Rev 2007; 24: 117–128. 18059629 10.1080/02648725.2007.10648095

[bib29] Lange H. Comparative test of methods to determine particle size and particle size distribution in the submicron range. Part Part Syst Char 1995; 12: 148–157.

[bib30] Mahl D, Diendorf J, Meyer-Zaika W, Epple M. Possibilities and limitations of different analytical methods for the size determination of a bimodal dispersion of metallic nanoparticles. Colloids Surf A 2011; 377: 386–392.

[bib31] Hassan PA, Kulshreshtha SK. Modification to the cumulant analysis of polydispersity in quasielastic light scattering data. J Colloid Interface Sci 2006; 300: 744–748. 16790246 10.1016/j.jcis.2006.04.013

[bib32] Piliarik M, Sandoghdar V. Direct optical sensing of single unlabelled proteins and super-resolution imaging of their binding sites. Nat Commun 2014; 5: 4495. 25072241 10.1038/ncomms5495

[bib33] Gaiduk A, Ruijgrok PV, Yorulmaz M, Orrit M. Detection limits in photothermal microscopy. Chem Sci 2010; 1: 343–350.

[bib34] Wu YC, Shiledar A, Li YC, Wong J, Feng S et al. Air quality monitoring using mobile microscopy and machine learning. Light Sci Appl 2017; 6: e17046, doi:10.1038/lsa.2017.46. 10.1038/lsa.2017.46 PMC6062327 30167294

[bib35] Vollmer F, Braun D, Libchaber A, Khoshsima M, Teraoka I et al. Protein detection by optical shift of a resonant microcavity. Appl Phys Lett 2002; 80: 4057–4059.

[bib36] Zhu JG, Özdemir ŞK, Xiao YF, Li L, He LN et al. On-chip single nanoparticle detection and sizing by mode splitting in an ultrahigh-*Q* microresonator. Nat Photonics 2010; 4: 46–49.

[bib37] Zhi YY, Yu XC, Gong QH, Yang L, Xiao YF. Single nanoparticle detection using optical microcavities. Adv Mater 2017; 29: 1604920. 10.1002/adma.201604920 28060436

[bib38] Yu WY, Jiang WC, Lin Q, Lu T. Cavity optomechanical spring sensing of single molecules. Nat Commun 2016; 7: 12311. 27460277 10.1038/ncomms12311 PMC4974467

[bib39] Swaim JD, Knittel J, Bowen WP. Detection of nanoparticles with a frequency locked whispering gallery mode microresonator. Appl Phys Lett 2013; 102: 183106.

[bib40] Sumetsky M, Windeler RS, Dulashko Y, Fan X. Optical liquid ring resonator sensor. Opt Express 2007; 15: 14376–14381. 19550715 10.1364/oe.15.014376

[bib41] Wiersig J. Enhancing the sensitivity of frequency and energy splitting detection by using exceptional points: application to microcavity sensors for single-particle detection. Phys Rev Lett 2014; 112: 203901.

[bib42] Liu ZP, Zhang J, Özdemir ŞK, Peng B, Jing H et al. Metrology with PT-symmetric cavities: enhanced sensitivity near the PT-Phase transition. Phys Rev Lett 2016; 117: 110802. 27661674 10.1103/PhysRevLett.117.110802

[bib43] Heylman KD, Thakkar N, Horak EH, Quillin SC, Cherqui C et al. Optical microresonators as single-particle absorption spectrometers. Nat Photonics 2016; 10: 788–795.

[bib44] Su J, Goldberg AFG, Stoltz BM. Label-free detection of single nanoparticles and biological molecules using microtoroid optical resonators. Light Sci Appl 2016; 5: e16001, doi:10.1038/lsa.2016.1. 10.1038/lsa.2016.1 PMC6059845 30167109

[bib45] Yu XC, Li BB, Wang P, Tong LM, Jiang XF et al. Single nanoparticle detection and sizing using a nanofiber pair in an aqueous environment. Adv Mater 2014; 26: 7462–7467. 25164067 10.1002/adma.201402085

[bib46] Xu F, Brambilla G. Embedding optical microfiber coil resonators in Teflon. Opt Lett 2007; 32: 2164–2166. 17671571 10.1364/ol.32.002164

[bib47] Xu F, Brambilla G. Demonstration of a refractometric sensor based on optical microfiber coil resonator. Appl Phys Lett 2008; 92: 101126.

[bib48] Wang SS, Pan XY, Tong LM. Modeling of nanoparticle-induced Rayleigh-Gans scattering for nanofiber optical sensing. Opt Commun 2007; 276: 293–297.

[bib49] Zhu JG, Özdemir ŞK, Yang L. Optical detection of single nanoparticles with a subwavelength fiber-taper. IEEE Photonics Technol Lett 2011; 23: 1346–1348.

[bib50] Salas-Montiel R, Apuzzo A, Delacour C, Sedaghat Z, Bruyant A et al. Quantitative analysis and near-field observation of strong coupling between plasmonic nanogap and silicon waveguides. Appl Phys Lett 2012; 100: 231109.

[bib51] Petersen J, Volz J, Rauschenbeutel A. Chiral nanophotonic waveguide interface based on spin-orbit interaction of light. Science 2014; 346: 67–71. 25190718 10.1126/science.1257671

[bib52] Tong LM, Lou JY, Mazur E. Single-mode guiding properties of subwavelength-diameter silica and silicon wire waveguides. Opt Express 2004; 12: 1025–1035. 19474918 10.1364/opex.12.001025

[bib53] Swaim JD, Knittel J, Bowen WP. Tapered nanofiber trapping of high-refractive-index nanoparticles. Appl Phys Lett 2013; 103: 203111.

[bib54] Tong LM, Gattass RR, Ashcom JB, He SL, Lou JY et al. Subwavelength-diameter silica wires for low-loss optical wave guiding. Nature 2003; 426: 816–819. 14685232 10.1038/nature02193

[bib55] Brambilla G, Finazzi V, Richardson DJ. Ultra-low-loss optical fiber nanotapers. Opt Express 2004; 12: 2258–2263. 19475062 10.1364/opex.12.002258

[bib56] Mauranyapin NP, Madsen LS, Taylor MA, Waleed M, Bowen WP. Evanescent single-molecule biosensing with quantum-limited precision. Nat Photonics 2017; 11: 477–481.

[bib57] Goban A, Choi KS, Alton DJ, Ding D, Lacroûte C et al. Demonstration of a state-insensitive, compensated nanofiber trap. Phys Rev Lett 2012; 109: 033603. 22861848 10.1103/PhysRevLett.109.033603

[bib58] Gouraud B, Maxein D, Nicolas A, Morin O, Laurat J. Demonstration of a memory for tightly guided light in an optical nanofiber. Phys Rev Lett 2015; 114: 180503. 26000992 10.1103/PhysRevLett.114.180503

[bib59] Reitz D, Sayrin C, Mitsch R, Schneeweiss P, Rauschenbeutel A. Coherence properties of nanofiber-trapped cesium atoms. Phys Rev Lett 2013; 110: 243603. 25165922 10.1103/PhysRevLett.110.243603

[bib60] Ren JJ, Gu Y, Zhao DX, Zhang F, Zhang TC et al. Evanescent-vacuum-enhanced photon-exciton coupling and fluorescence collection. Phys Rev Lett 2017; 118: 073604. 28256881 10.1103/PhysRevLett.118.073604

[bib61] Blaize S, Gesuele F, Stefanon I, Bruyant A, Lérondel G et al. Real-space observation of spectral degeneracy breaking in a waveguide-coupled disk microresonator. Opt Lett 2010; 35: 3168–3170. 20890322 10.1364/OL.35.003168

[bib62] Apuzzo A, Février M, Salas-Montiel R, Bruyant A, Chelnokov A et al. Observation of near-field dipolar interactions involved in a metal nanoparticle chain waveguide. Nano Lett 2013; 13: 1000–1006. 23413879 10.1021/nl304164y

[bib63] Stefanon I, Blaize S, Bruyant A, Aubert S, Lérondel G et al. Heterodyne detection of guided waves using a scattering-type scanning near-field optical microscope. Opt Express 2005; 13: 5553–5564. 19498552 10.1364/opex.13.005553

[bib64] Robinson JT, Preble SF, Lipson M. Imaging highly confined modes in sub-micron scale silicon waveguides using transmission-based near-field scanning optical microscopy. Opt Express 2006; 14: 10588–10595. 19529461 10.1364/oe.14.010588

[bib65] Gomez L, Bachelot R, Bouhelier A, Wiederrecht GP, Chang SH et al. Apertureless scanning near-field optical microscopy: a comparison between homodyne and heterodyne approaches. J Opt Soc Am B 2006; 23: 823–833.

