# Supplementary material for: Optically sizing single atmospheric particulates with a 10-nm resolution using a strong evanescent field
Source: Light Sci Appl. 2018 Apr 20;7:18003–. doi: 10.1038/lsa.2018.3 (PMC6060056; doi:10.1038/lsa.2018.3)
Supplement: Supplementary Information [file lsa20183x1.pdf]

Supporting Materials - Optically sizing single atmospheric particlulates with a 10-nm resolution using strong evanescent field

Xiao-Chong Yu,[1] Yanyan Zhi,[1] Shui-Jing Tang,[1,2] Bei-Bei Li,[1] Qihuang Gong,[1,2] Cheng-Wei Qiu,[3,4,5] Yun-Feng Xiao[1,2]

[1]State Key Laboratory for Mesoscopic Physics and School of Physics, Peking University; Collaborative Innovation Center of Quantum Matter, Beijing 100871, People's Republic of China

[2]Collaborative Innovation Center of Extreme Optics, Taiyuan 030006, Shanxi, People's Republic of China

[3]Department of Electrical and Computer Engineering, National University of Singapore, 4 Engineering Drive 3, Singapore 117583, Singapore

[4]NUS Suzhou Research Institute (NUSRI), Suzhou Industrial Park, Suzhou 215123, People's Republic of China

[5]SZU-NUS Collaborative Innovation Center for Optoelectronic Science & Technology, Shenzhen University, Shenzhen 518060, People's Republic of China

In Section I, we discuss how to calculate the scattering efficiency induced by a single nanoparticle, through both Rayleigh-Gans scattering theory and three-dimensional finite-element-method (3D FEM) simulation. In addition, we discuss the interference effect between particles. In Section II, we show how to atomize the standard polystyrene nanoparticles and generate single particles from the solution. The sizing uncertainty of the air particulate matters, resulting from the difference of their refractive indices, is also discussed. Section III provides the details of fabricating the nanowaveguide structure. In Section IV, we describe the data acquisition and analysis methods, and the source of the official data is also included.

I. Theoretical analysis and numerical calculations of nanowaveguide-particle interaction

A. Rayleigh-Gans scattering theory

In this section, we calculate the scattering efficiency induced by a single nanoparticle bound on the nanowaveguide surface, using the Rayleigh-Gans scattering theory. A nanoparticle with excess polarizability χ will be polarized, when it locates in an electric field with the strength of $\vec{E}_1$. The polarization density is described as $\vec{P}_2 = \varepsilon_0 \chi \vec{E}_1$. The total radiation from the scatterer can be calculated as superposition of all dipole emitters inside the whole volume, and the far-field electromagnetic fields are

$$\vec{E} = -\frac{\mu_0}{4\pi R}\frac{\vec{R}}{R}\times\left[\frac{\vec{R}}{R}\times\int_V \vec{P}_2 e^{-ik\frac{\vec{R}}{R}\cdot\vec{r}}dV\right], \quad \text{(S1)}$$

$$\vec{H} = \frac{\omega^2}{4\pi cR}\frac{\vec{R}}{R}\times\int_V \vec{P}_2 e^{-ik\frac{\vec{R}}{R}\cdot\vec{r}}dV, \quad \text{(S2)}$$

where V is over the whole volume of the nanoparticle, ω denotes the angular frequency of the probe light, μ_0 is the permeability of vacuum, $\vec{R}$ is the far-field position vector with the norm R, and $\vec{r}$ the position vector inside the nanoparticle.

The far-field radiation can be calculated by

$$S(\theta,\varphi)=\frac{\mu_0\omega^2}{32\pi^2}\left|\hat{R}\times\int_V \vec{P}_2 e^{-ik\hat{R}\cdot\vec{r}}dV\right|^2, \tag{S3}$$

where θ and φ are the angular and azimuthal angles, respectively, and $\hat{R}=\vec{R}/R$. The scattering efficiency is therefore

$$P_{\text{sca,RG}}=\oint S(\theta,\varphi)\sin^2\theta d\theta d\varphi . \tag{S4}$$

The size of the nanoparticle can then be derived from the scattering efficiency, for a given refractive index of the nanoparticle. The mass concentration of the nanoparticles can be further obtained, for a given density. Since the main components of the particulate matters are sulfates, nitrates and ammonium salt (1), the refractive index and the density of nanoparticles are set to be 1.5 and 2 g/cm^3 in the calculation (see also, Sec. II-B of this supporting information).

Using this method, the scattering efficiency as functions of the nanowaveguide diameter (with circular cross-sections) and nanoparticle diameter is plotted in Fig. 2 in the main text. The sizes of the nanoparticles are also calculated using the Rayleigh-Gans theory in Fig. 3c, Fig. 4, and Fig. 5 in the main text.

B. **Three-dimensional finite-element-method simulation**

A 3D FEM simulation is also used to calculate the scattering efficiency, to confirm the results obtained from Rayleigh-Gans scattering theory. Here, to balance the accuracy and resource consumption, we use the reciprocity theorem instead of the direct S-parameter calculation using port,

$$\oint_S\left(\vec{E}_1^*\times\vec{H}_2+\vec{E}_2\times\vec{H}_1^*\right)\cdot\vec{dA}=-\int_V\left(\vec{J}_1^*\cdot\vec{E}_2+\vec{E}_1^*\cdot\vec{J}_2\right)dV \tag{S5}$$

Here, the electric field $\vec{E}_{1,2}$, magnetic field $\vec{H}_{1,2}$ and current density $\vec{J}_{1,2}$ are two set of stable solutions of electromagnetic field, labeled as 1 and 2, sharing the same geometry. For the nanowaveguide system, ($\vec{E}_1$, $\vec{H}_1$, $\vec{J}_1=0$) represents the field for the fundamental modes, and ($\vec{E}_2$, $\vec{H}_2$, $\vec{J}_2$) is the field with the existence of the nanoparticle, where $\vec{J}_2=-i\omega\vec{P}_2$ is the polarizing current density inside the scatterers. Note that, the electric field of the fundamental modes for nanowaveguide includes both the transverse and longitudinal parts.

The electromagnetic field can be expanded using the guided modes and the radiation modes, and only the guided modes can be detected as the transmission and reflection. The scattering parameter between the guided modes μ and ν can be written as

$$S_{\mu\nu}=-\frac{\int_V \vec{E}_\mu^*\cdot\vec{J}_\nu dV}{2\,\mathrm{Re}\int_S \vec{E}_\mu^*\times\vec{H}_\mu\cdot dA}, \tag{S6}$$

with the integral area S perpendicular to the waveguide. For spherical nanoparticles, the vertically and horizontally polarized modes have no cross-talk due to the symmetry, and thus can be considered separately. The circularly polarized mode can be treated by averaging the scattering and reflection of the two linearly polarized modes. By applying the polarizing current density $\vec{J}_\nu$ inside the particle and the boundary mode ($\vec{E}_\mu$, $\vec{H}_\mu$) of the nanowaveguide using 3D FEM results, the scattering efficiency $P_{\text{sca,3D}}=1-\left|1+S_{11}\right|^2$ and the reflection efficiency $R=\left|S_{12}\right|^2$ can thus be obtained. The comparison of the scattering efficiency calculated using Rayleigh-Gans scattering theory and the 3D FEM simulation is

plotted in Fig. 2a of the main text. The integration volume can also be non-continuous, so that the same approach can be used to calculate the multi-particle scattering case.

C. Polarizations of the probe light

In theory, the particle scattering depends strongly on the polarization of the evanescent field around the waveguide. Using Rayleigh-Gans scattering theory and 3D FEM simulation, we calculate the scattering efficiency induced by a single nanoparticle bounded on the nanowaveguide surface, for probe light with different polarizations. Figure S1A shows the results of three different polarizations: horizontal polarization, vertical polarization, and circular polarization. As discussed in the main text, the scattering efficiency P_{sca}, as a function of the particle size, is highly dependent on the mode polarizations. For example, due to the strongly inhomogeneous field distributions for the two linearly polarized modes (Fig. S1B, i-ii), the scattering efficiencies of the horizontally and vertically modes are $P_{sca}^{V} = 1.14\%$ and $P_{sca}^{H} = 0.31\%$, respectively, for a particle with a diameter of 200 nm. For an arbitrarily linear polarization, the scattering efficiency can be calculated from the superposition of that of the two orthogonal polarizations $P_{sca} = P_{sca}^{V} \cos^2\theta + P_{sca}^{H} \sin^2\theta$. This means even for one specific probe light with linear polarization, the scattering efficiency is not identical due to the inhomogeneity of the field distribution, when the particle binds to different positions around the nanowaveguide surface. The uncertainty on P_{sca} induced by field inhomogeneity adds to that of the particle sizing measurements. The differences on P_{sca} resulting from the field inhomogeneity can be avoided by using a circularly polarized mode, of which the cross-sectional field distribution is homogeneous around the waveguide boundary, and the scattering efficiency $P_{sca} = \left(P_{sca}^{V} + P_{sca}^{H}\right)/2$ shows independence on the particle locations (Fig. S1B, iii).

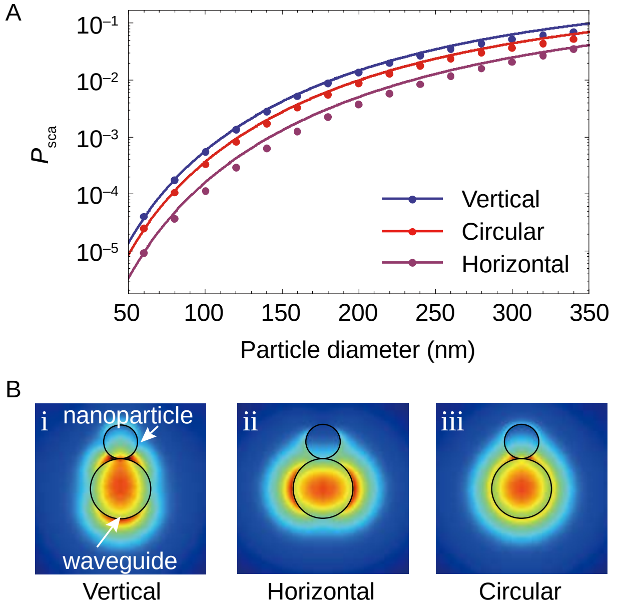

Fig. S1. (**A**) The scattering efficiency as a function of particle diameter calculated by Rayleigh-Gans theory (solid curves) and 3D FEM simulation (symbols), when the probe light is vertically, horizontally and circularly polarized. The diameter of the nanowaveguide is 350 nm. The refractive indices of the nanowaveguide and the nanoparticle are 1.46 and 1.5, respectively. (**B**) 3D FEM simulation showing the cross-section of the electric field distribution of the vertical, horizontal and circular polarized probe light, when a 200-nm-diameter PS particle attaches to a 350-nm-diameter nanowaveguide.

D. **Interference induced by particles**

Multiple nanoparticles on the nanowaveguide may cause interference through both the guided modes and the radiation modes. To study this interference, we consider two identical nanoparticles (with a diameter of 200 nm) on a nanowaveguide (with a diameter of 350 nm), using a circularly polarized probe light. Using the 3D FEM simulations, Fig. S2A shows the scattering efficiency when the two nanoparticles locate at the same and opposite sides of the nanowaveguide, as a function of the distance between two particles. Evident oscillations are observed due to mode interference, as expected. However, when the distance between the two particles is larger than 2 μm, the oscillation becomes weak, and the difference in P_{sca} induced by particle locations is less than 5%, suggesting a high accuracy in single particle sizing measurements.

One may argue that the reflection efficiency can also be used as a sensing signal. Here, we calculate the refection as a function of the two-particle distance. Compared to the scattering signal, the reflection magnitude is much smaller and with strong oscillations as a function of the two-particle distance (shown in Fig. S2B), indicating that the reflection cannot be used as a reasonable sensing signal for nanoparticle sizing.

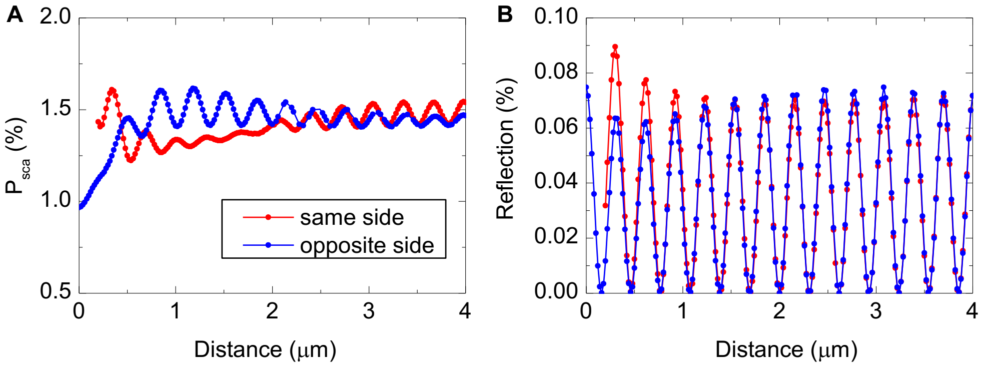

Fig. S2. Scattering (**A**) and reflection (**B**) efficiencies induced by two 200-nm-diameter nanoparticles on a 350-nm-diameter nanowaveguide, as a function of the distance between them, using 3D FEM simulation. The probe light is circularly polarized. The red (blue) curves indicate the case when the two particles locate on the same (opposite) side around the nanowaveguide, respectively.

II. **Standard particles and air particulates**

A. **Standard particle atomization**

The polystyrene (PS) particles are atomized to be single nanoparticles using an ultrasonic atomizer. The droplet size is dependent on the wavelength of the capillary wave, and the median droplet size can be calculated as

$$D = 0.34\sqrt[3]{\frac{8\pi\sigma}{\rho f^2}}, \qquad \text{(S7)}$$

where σ is the surface tension, ρ is the density of the liquid, and f is the exciting sound frequency (2). In the experiment, $f = 1.7$ MHz, $\sigma = 7.2 \times 10^{-2}$ N/m, $\rho = 1000$ kg/m^3, thus $D = 3$ μm. The concentration of the standard PS nanoparticles used in the experiments is about 2 pM, so the average number of particles in each droplet is calculated to be as low as 0.015, ensuring one single nanoparticle in one droplet. When blown onto the waveguide, the liquid in the droplet is vaporized, ensuing single nanoparticle binding events. In order to confirm this, the atomized 90-nm-diameter nanoparticles are

blown onto a silicon substrate, and the scanning electron microscope (SEM) image shown in Fig. S3 shows a single nanoparticle distribution.

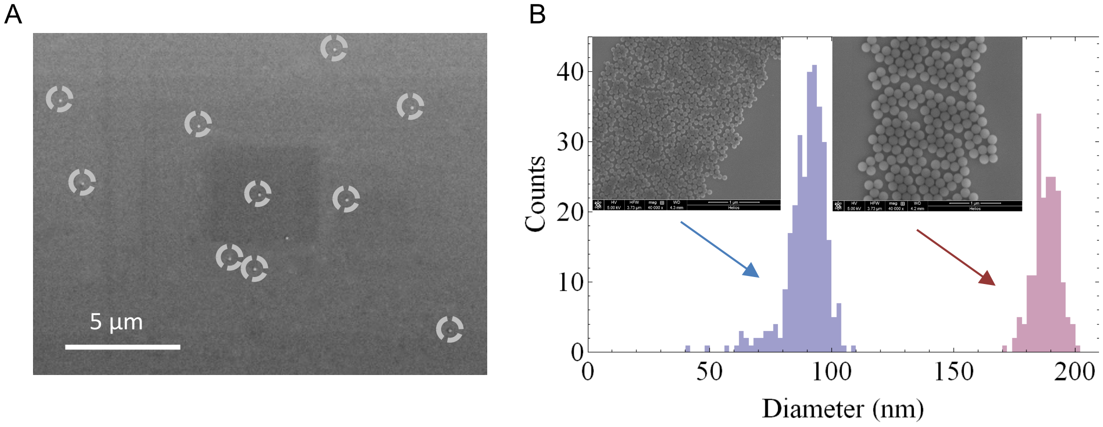

Fig. S3. (**A**) SEM image of the atomized PS nanoparticles on a silicon substrate. The bright dots labeled by dashed circle indicate single particles. (**B**) Size distribution of two standard nanospheres with mean diameters of 89.6±8.7 nm and 188.0±5.2 nm. Inset: SEM image of two samples of nanospheres.

B. **Particle refractive index**

For nanoparticles with different refractive indices, the scattering efficiency calculated by Rayleigh-Gans theory is proportional to $\left(n_p^2-1\right)^2$. When calculating the particulate size, the particulate refractive index is set as 1.5 in the main text. This is reasonable, because the particulates in air are mostly sulfates, nitrates and ammonium salts with refractive indices ranging from 1.45 to 1.55 (1), which adds slight uncertainties to the sizing measurements. When a nanoparticle binds to a waveguide with a diameter of 350 nm, Fig. S4 shows scattering efficiency as functions of the particle diameter and the particle refractive index using 3D simulation. The sizing uncertainties for scattering efficiencies of 0.1%, 0.2%, 0.5%, 1%, 2% and 5% are listed in Table S1, which are less than 10%.

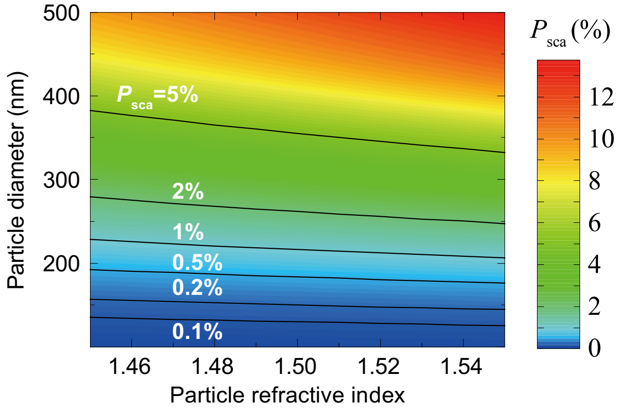

Fig. S4. Scattering efficiency as functions of the particle diameter and the particle refractive index, when a nanoparticle binds to a waveguide with a diameter of 350 nm. The solid curves indicate the scattering efficiencies of 5%, 2%, 1%, 0.5%, 0.2% and 0.1%.

Table S1 Sizing uncertainties for given scattering efficiencies, when the particle refractive index is 1.45 and 1.55 respectively.

P_{sca}	Particle refractive	Calculated particle diameter	Sizing
0.1%	1.45	135.2	±3.8%
	1.55	125.3	
0.2%	1.45	156.6	±4.0%
	1.55	144.5	
0.5%	1.45	192.2	±4.4%
	1.55	175.9	
1%	1.45	228.2	±5.0%
	1.55	206.5	
2%	1.45	279.0	±6.0%
	1.55	247.6	
5%	1.45	382.5	±7.1%
	1.55	332.3	

III. Nanowaveguide fabrication and diameter control

The nanowaveguide structure consists of five identical nanofibers. The nanofiber drawing process can be described using the thin filament equations considering the mass conservation and the force balance (3,4),

$$\begin{cases} A_t + (uA)_x = 0 \\ (3\mu A u_x)_x = 0 \end{cases}, \qquad \text{(S8)}$$

where $A(x, t)$ is the fiber cross-section area as a function of the fiber position and the pulling time, u denotes the pulling speed, and μ represents the coefficient of viscosity. By separating the spatial and time dependent parts of $A(x, t)$,

$$-\frac{A_t}{uA} = \frac{A_x}{A}\frac{\int_0^x \frac{dx}{\mu A}}{\int_0^\infty \frac{dx}{\mu A}} + \frac{\frac{1}{\mu A}}{\int_0^\infty \frac{dx}{\mu A}} = C, \qquad \text{(S9)}$$

where C is a constant, the shape of a nanofiber can be predicted, and the diameter of the nanofiber can be controlled by the pulling time. To verify this prediction, a nanofiber is fabricated, of which the diameters are measured from the SEM images. The diameter distribution of the nanofiber is consistent with the theoretical predictions using the parabolic temperature distribution of the heater, as shown in Fig. 1c in the main text. Note that the nanofiber only has a 10% variation in diameter within 3 millimeters at the central region, reducing the uncertainty on sizing the target nanoparticles via the sensing measurements. The minimum diameter, i.e., at the central point of the nanofiber, can also be calculated from numerical solutions of Eq. S8.

IV. Data acquisition and noise analysis

A. Data acquisition

The transmission power collected by the photodetector (Newport, Model 918D) is analyzed by a data acquisition system (National Instrument, Model USB-6251 BNC) with an acquisition rate of 100 kS/s.

In this case, only the binding events are detectable, because the typical time for particulates bouncing off and passing through nanofiber is far less than one microsecond. In order to decrease the noise level of the transmission power to resolve smaller steps and thus smaller nanoparticles, a 2000-point average is applied, which suppresses the noise level from 10^{-3} to about 10^{-4}, as shown in a typical transmission signal in Fig. S5. After the noise suppression, the total detectable events for the nanoparticles with scattering efficiency at 10^{-3} level can easily exceed one thousand. A step finding algorithm[S2] is applied to determine the step drops in the transmission signal, which corresponds to the single nanoparticle binding events. The transmission steps shown in Fig. 3a in the main text are pro-processed in this method.

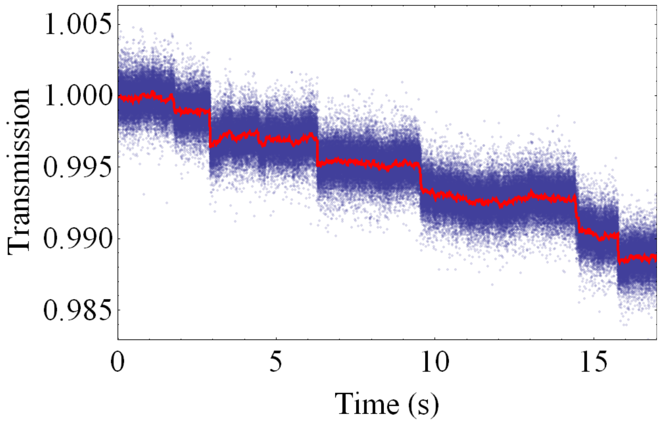

Fig. S5. Raw data captured by the data acquisition system (blue dots) and the processed data using a 2000-points averaging (red curve). All data are normalized by the first points of the post-processed data.

Figure S6 shows the transmission signals induced by same single nanoparticles with diameters of 130 nm, and mixed single nanoparticles with diameters of 130 nm and 200 nm, respectively. The steps induced by particles with the same diameters are quite uniform in Fig. S6A (and Fig. 3a in the main text), while two sets of steps with different magnitude are observed in Fig. S6B (and Fig. 3c in the main text for three types of nanoparticles) due to distinct scattering efficiencies induced by particles with varied diameters, as expected. Note that, the increase step in Fig. S6B indicates the nanoparticle escape.

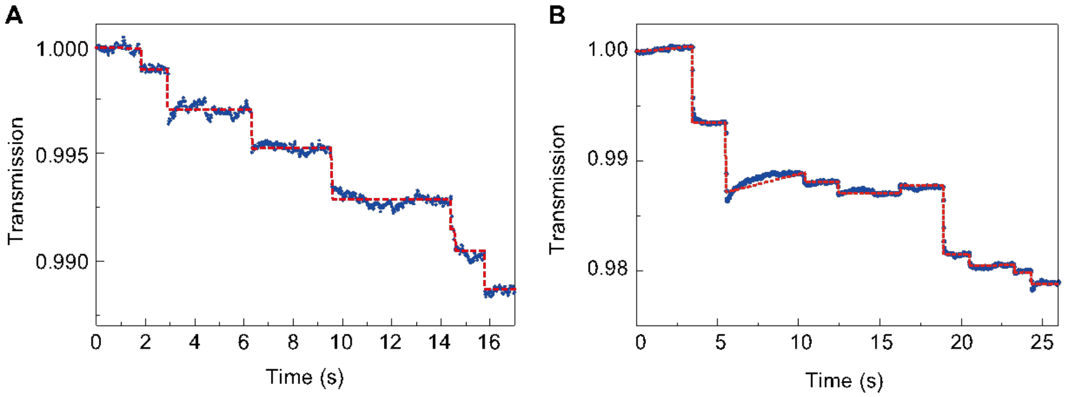

Fig. S6. Transmission signals induced by same single nanoparticles with diameters of 130 nm (**A**) and

by mixed single nanoparticles with diameters of 130 nm and 200 nm (**B**).

B. **Noise analysis**

The nanoparticle counts for a small size range of Δr are considered to follow the Poisson distribution with a mean value of $\eta(r)p(r)V\Delta r$, where $\eta(r)$ is the capturing probability, $p(r)$ is the number concentration of the particle with radius r and V is the total sample volume. The distribution of the total counts N can thus be calculated as a Poisson distribution with a mean value of $\overline{N} = \sum \eta(r)p(r)V\Delta r = \int \sum \eta(r)p(r)Vdr$. The standard deviation of N is $\sqrt{\overline{N}}$.

Now, considering the mass concentration, the mean value of the total capturing mass $\overline{M} = \int \sum \eta(r)m(r)p(r)Vdr$, and the standard deviation $\Delta M = \sqrt{\int \eta(r)m^2(r)p(r)Vdr}$, where $m(r)$ is the mass of one single nanoparticle. This is also one kind of the shot noises. The further consideration of the single nanoparticle mass distribution gives $\Delta M = \sqrt{\int \eta(r)[\overline{m}^2(r) + \Delta m^2(r)]p(r)Vdr}$, where $\overline{m}$ and Δm is the mean value and standard deviation of the particle mass respectively. The additional term corresponds to the uncertainty on particle material and the noise on measurement. As expected, ΔM has a stronger dependence on larger particles, and obviously, $\Delta M / \overline{M} \propto 1/\sqrt{V}$. For both the number concentration and the mass concentration, the most effective way to suppress the uncertainty is to increase the capture probability and the volume of the sample air.

In the time domain, for a specific counting rate p_t, the periods between two nearby binding events obey the exponential distribution $p(t) = p_t e^{-p_t t}$. And for the smallest resolvable time Δt, the multi-particle binding probability can be calculated as $p_t \Delta t$. In our experiment, Δt is 20 ms, $p_t < 1\ s^{-1}$, the possibility of the multi-particle events is less than 2%.

C. **Capture ability**

To compare the capture ability, we detect the standard nanospheres at the same concentration utilizing single nanofiber, nanofiber pair and nanofiber array, respectively. (Fig. S7) Then we analyze the time interval between two nearby binding events, and fit the data using the exponential distribution. The mean time interval is about 12.2 s, 7.8 s and 4.0 s, respectively. It shows that the fiber array significantly enhances the capture ability.

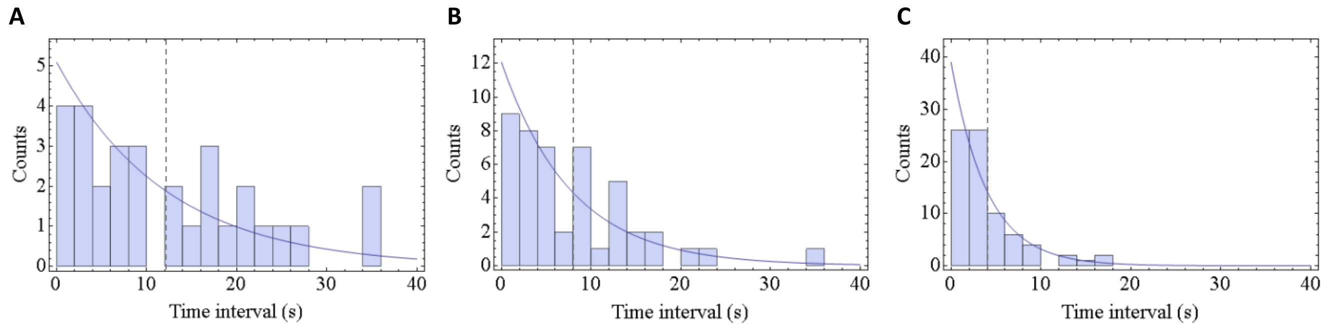

Fig. S7. Statistical analysis of the time intervals between two sequential nanoparticle binding events

when the detection system contains one nanofiber (**A**), two nanofibers (**B**), and five nanofibers (**C**).

D. **Additional experimental data**

During the first red alert for air pollution in 2016 in Beijing (from December 25th to 26th, 2016), we measure the urban air sample nearly every hour. There is an obvious decrease in both the mass concentration and size distribution (Fig. S8) after 3am at December 26th, which is in good agreement with the official data. The counts for particle above 400 nm in diameter approaches zero after 3am at December 26th (Fig. S8A), which contribute to most part of the mass concentration (Fig. S8B). The evolution trend of the particulate concentration agrees with the officially issued data (Fig. S8C).

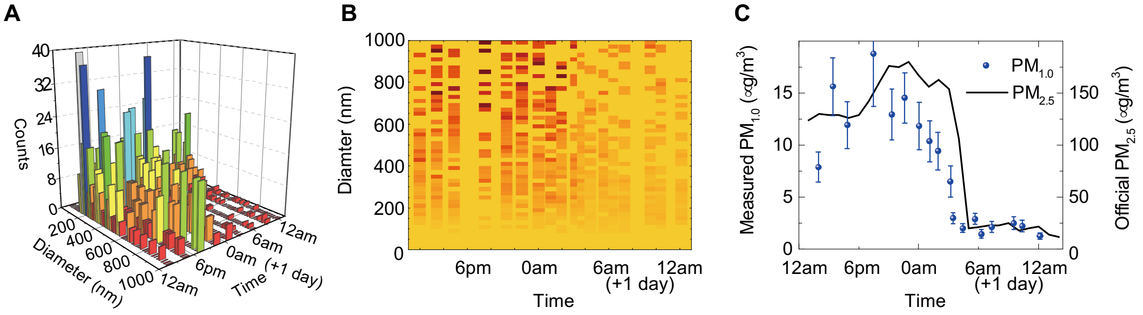

Fig. S8. Urban air monitoring during the first red alert for air pollution in 2016 in Beijing (from December 25th to 26th, 2016). (**A**) Size histogram of nanoparticles in nine air samples collected from different time point. (**B**) The evolution of the mass concentration of the nanoparticles of different diameters with a 20 nm step. (**C**) The evolution of the measured $PM_{1.0}$ index (blue spheres, left axis), compared with the officially reported $PM_{2.5}$ index from BJMEMC (solid curve, right axis), error bars indicate the standard deviation.

E. **Official data**

The urban environment air sample in the experiment is collected at School of Physics, Peking University (Latitude 39.992°, Longitude 116.316°). The measured data is compared with the official data reported by Beijing Municipal Environmental Monitoring Center (BJMEMC, http://zx.bjmemc.com.cn/) to evaluate the capability of the size spectrometer. The official data in Fig. S9 shows the $PM_{2.5}$ mass concentration reported every hour from December 9th, 2015 to January 12th, 2016, and the air samples are from the closest official site, Wanliu, Haiden district (Latitude 39.987°, Longitude 116.287°).

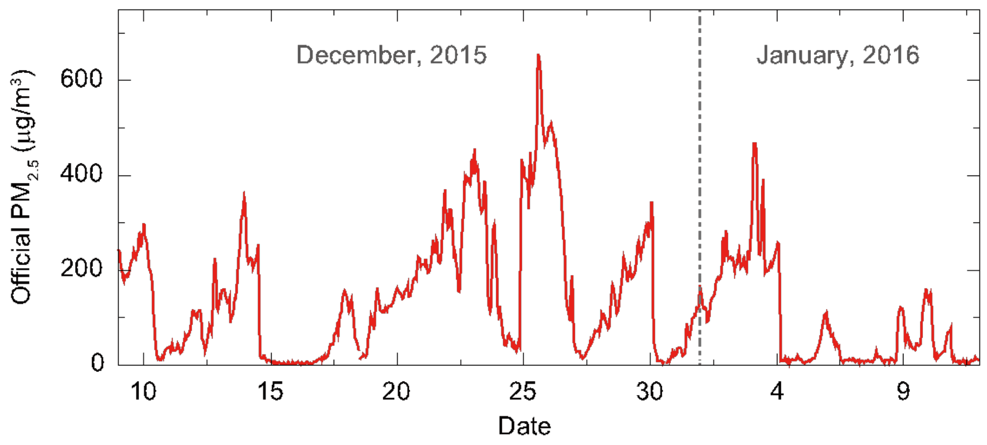

Fig. S9. Official $PM_{2.5}$ data from Wanliu, reported every hour, continuously from December 9th, 2015 to January 12th, 2016.

References

1. Huang RJ, et al. (2014) High secondary aerosol contribution to particulate pollution during haze events in china. Nature 514(7521):218–22.
2. Lang RJ (1962) Ultrasonic atomization of liquids. J. Acoust. Soc. Am. 34(1):6–8.
3. Dewynne J, Ockendon JR, Wilmott P (1989) On a mathematical-model for fiber tapering. Siam J Appl Math 49(4):983–990.
4. Sørensen HL, Polzik ES, Appel J (2014) Heater self-calibration technique for shape prediction of fiber tapers. J. Lightwave Technol. 32(10):1886–1891.
